# 17β-estradiol and methylation of migraine-related genes

**DOI:** 10.1186/1129-2377-14-S1-P29

**Published:** 2013-02-21

**Authors:** S Labruijere, M Verbiest, R De Vries, AHJ Danser, AG Uitterlinden, L Stolk, A MaassenVanDenBrink

**Affiliations:** 1 Erasmus Medical Center, Netherlands

## 

Migraine is much more common in women than in men, especially during the reproductive part of their lives. Fluctuations in the female hormone 17β-estradiol throughout the menstrual cycle are thought to be an important factor in causing migraine attacks in women. Although it has been shown that 17β-estradiol can change vascular sensitivity to CGRP, it is still unknown how 17β-estradiol causes these effects. GWAS studies have identified genes that may be relevant for migraine, but an influence of environmental factors is likely. Interestingly, the prophylactic effectivity of valproate, a DNA methylation inhibitor, may point to the involvement of epigenetic mechanisms in migraine. 17β-estradiol is known to be involved in epigenetic mechanisms and recently it was shown that CGRP can be regulated via epigenetic mechanisms. Therefore, the aim of this study was to investigate the role of 17β-estradiol in the methylation of migraine-related genes. Female SD rats were ovariectomized (ovx) and treated with 17β-estradiol or placebo pellets. DNA was isolated from blood, aorta, dura mater, trigeminal caudal nucleus and trigeminal ganglion. PCR was performed for 10 migraine- and female hormone-related genes (MTHFR, eNOS, ESR1, GPER, CGRP, USF1, USF2, RAMP1, CRCP and CRLR) and DNA methylation was assessed through bisulfite treatment and sequenom mass spectrometry. No difference in DNA methylation was seen between control animals, ovx placebo-treated or ovx 17β-estradiol-treated animals for the MTHFR, GPER, eNOS, CGRP, USF1 and USF2 genes. Tissue-specific differences in methylation were seen for the ESR1, CRCP, eNOS and CRLR genes. Remarkably, our results point to an increase in methylation of the CRCP gene in the trigeminal caudal nucleus in the 17β-estradiol-treated animals. These esults indicate a possible epigenetic regulatory mechanism for one part of the CGRP receptor in the trigeminal caudal nucleus.
